# Imperatorin: A Furanocoumarin with Potential in Combating Cancer Development and Progression—A Comprehensive Review

**DOI:** 10.3390/ph19030436

**Published:** 2026-03-08

**Authors:** Victória Dogani Rodrigues, Cláudia Rucco Penteado Detregiachi, Manuela dos Santos Bueno, Luíza Santos de Argollo Haber, Rachel Gomes Eleutério, Eliana de Souza Bastos Mazuqueli Pereira, Virgínia Maria Cavallari Strozze Catharin, Lidiane Indiani, Vitor Cavallari Strozze Catharin, Sérgio Zabotto Dantas, Kátia Portero Sloan, Caio Sergio Galina Spilla, Lance Alan Sloan, Karina Quesada, Sandra Maria Barbalho, Lucas Fornari Laurindo

**Affiliations:** 1 Department of Biochemistry and Pharmacology, School of Medicine, Faculdade de Medicina de Marília (FAMEMA), Marília 17519-030, SP, Brazil; 2 Graduate Program in Structural and Functional Interactions in Rehabilitation, School of Medicine, Universidade de Marília (UNIMAR), Marília 17525-902, SP, Brazil; 3 Department of Biochemistry and Pharmacology, School of Medicine, Universidade de Marília (UNIMAR), Marília 17525-902, SP, Brazil; 4 Department of Clinical Metabolism, Texas Institute for Kidney and Endocrine Disorders (TIKED), Lufkin, TX 75904, USA; 5 Department of Internal Medicine, University of Texas Medical Branch, Galveston, TX 77555, USA; 6 Division of Cellular Growth, Hemodynamics, and Homeostasis Disorders, Graduate Program in Medical Sciences, Faculdade de Medicina, Universidade de São Paulo (USP), São Paulo 01246-903, SP, Brazil

**Keywords:** imperatorin, phytochemicals, anticancer activity, apoptosis, cell cycle arrest, metastasis inhibition, chemoprevention, coumarins

## Abstract

Imperatorin, a naturally occurring furanocoumarin found in several medicinal plants, has attracted considerable scientific interest due to its broad spectrum of pharmacological activities and emerging relevance in oncology. In recent years, an increasing number of experimental studies have investigated its biological effects and molecular mechanisms across different tumor models. Due to this, the review synthesizes the current preclinical and pharmacological evidence on imperatorin in cancer, with the aim of consolidating the main mechanistic pathways involved in its antitumor activity, identifying its therapeutic opportunities, and highlighting existing challenges and future research perspectives. Available in vitro and in vivo studies demonstrate that imperatorin exerts multi-targeted antitumor effects, including the induction of apoptosis, inhibition of proliferation, suppression of angiogenesis, modulation of oxidative stress, attenuation of inflammation, and disruption of oncogenic signaling pathways such as PI3K/Akt, MAPK, mTOR, and NF-κB. Imperatorin also influences the tumor microenvironment by reducing pro-inflammatory mediators, impairing stromal–tumor cross-talk, and enhancing immune-cell-mediated cytotoxicity. In addition, we also summarize pharmacokinetic and safety limitations that hinder clinical translation, including low oral bioavailability, extensive plasma protein binding, cytochrome P450 interactions, and insufficient toxicological data. In parallel, we highlight recent advances in the genetics and biosynthesis of imperatorin, which support perspectives for sustainable production and structural optimization of imperatorin derivatives. Finally, we outline key knowledge gaps and future directions, including improved delivery strategies, investigation of additional regulatory pathways, and more robust in vivo and translational studies, emphasizing that imperatorin remains a promising yet still incompletely characterized anticancer candidate. The review highlights the need for more comprehensive pharmacokinetic and safety assessments, as well as the development of improved delivery systems to address absorption and stability challenges. Further research into imperatorin’s effects on autophagy, ferroptosis, metabolic reprogramming, and the immune microenvironment is essential to deepen mechanistic understanding. Additionally, fully elucidating the biosynthetic enzymes responsible for imperatorin formation may facilitate sustainable production and the design of structurally optimized analogs.

## 1. Introduction

Cancer is a heterogeneous disease characterized by uncontrolled cellular growth. Tumor development unfolds through several stages, beginning when a somatic cell acquires an oncogenic mutation that confers a proliferative advantage. As this altered cell divides, additional genetic and epigenetic changes accumulate, gradually driving the formation of tumors that become increasingly invasive and diverse in their cellular makeup. These progressive alterations fuel clonal expansion and accelerate cancer advancement. Environmental pressures and the natural effects of aging are major contributors to this process, as they disturb normal cellular homeostasis and create conditions that favor malignant transformation [1,2,3,4]. Within this context arises the tumor microenvironment—a dynamic milieu in which cancer cells coexist with a wide array of non-malignant cell types such as immune cells, fibroblasts, and endothelial cells, all embedded within a reorganized and vascularized extracellular matrix. The composition and behavior of this microenvironment vary widely depending on tumor type, anatomical site, disease stage, and individual patient factors. Importantly, its elements can both foster and restrain tumor growth, making it a critical determinant of cancer progression [5,6,7].

Cancer remains one of the most critical challenges in modern medicine, accounting for millions of new cases and deaths worldwide each year [8,9,10]. Despite advances in surgery, chemotherapy, radiotherapy, and targeted therapies, many cancers still present a poor prognosis due to drug resistance, severe side effects, high treatment cost, and relapse [11,12,13]. The need for novel, effective, and safer therapeutic agents remains urgent [14,15,16]. This urgency has prompted growing interest in alternative sources of anticancer agents, particularly natural products that offer both structural diversity and therapeutic potential.

**Figure 1:**
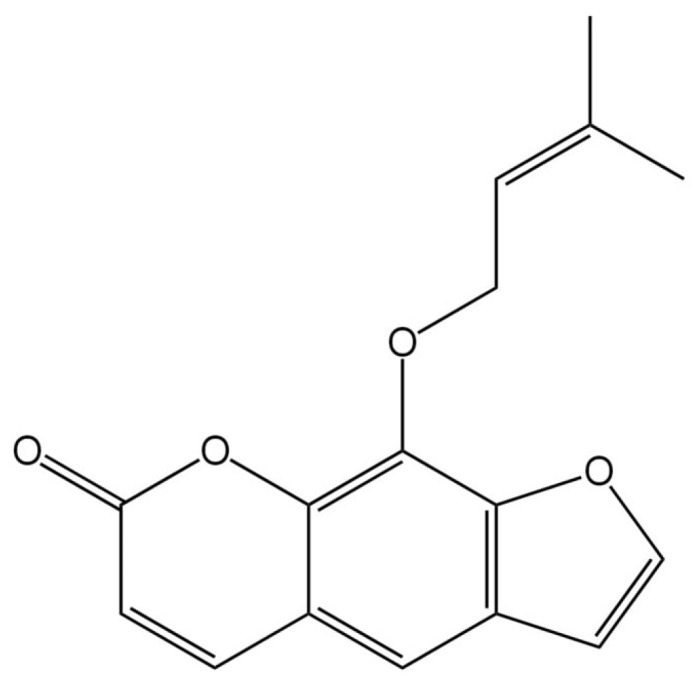
Molecular structure of imperatorin [32,33].

In recent decades, natural products—especially plant-derived phytochemicals—have gained renewed interest in oncology, driven by their structural diversity, biological activity, and often favorable safety profile [17,18,19,20,21]. Indeed, several clinically useful anticancer drugs originate from plants [22,23,24,25]. Among phytochemicals, coumarins and their derivatives stand out for their broad array of biological effects, including anti-inflammatory [26], antioxidant [27], neuroprotective [28], and anticancer properties [29]. One such promising compound is imperatorin, a furanocoumarin found in roots and other parts of medicinal plants such as *Angelica dahurica* (*Umbelliferae*) and various species of the *Rutaceae* and *Apiaceae* families. Traditionally used in herbal medicine for ailments such as inflammation, pain, and cardiovascular disorders, imperatorin’s broad pharmacological profile has spurred scientific interest beyond its classical uses [30,31]. Figure 1 depicts the molecular structure of imperatorin [32,33].

Emerging experimental evidence points to imperatorin’s potential as an anticancer agent. In vitro studies have demonstrated that imperatorin can inhibit proliferation and induce apoptosis in a variety of human cancer cell lines, including gastric cancer, colon cancer, and hepatoma, among others. The mechanisms implicated include mitochondrial (intrinsic) apoptosis via cytochrome c release and caspase activation, activation of pro-apoptotic regulators like p53, modulation of critical cancer-associated signaling pathways, and induction of cell cycle arrest. Additionally, imperatorin has been shown to suppress tumor growth in animal models [34,35,36]. Further studies reveal its ability to impair angiogenesis and tumor adaptation to hypoxia by inhibiting the synthesis of key factors, such as HIF-1α, and downregulating pathways like mTOR and MAPK—underscoring its multifaceted anticancer potential [37]. 

Beyond its pro-apoptotic and cell cycle regulatory effects, several studies have also explored other molecular targets underlying imperatorin’s pharmacological activity, which could be incorporated into cancer treatment studies. Screening-based and target-oriented investigations have reported inhibition of PDE4, resulting in increased intracellular cAMP levels and modulation of the PKA signaling axis in human neutrophils, which inhibits neutrophil respiratory burst, adhesion, and migration [38]. Although the cited study focuses on inflammatory skin disease, its demonstration that imperatorin selectively inhibits PDE4 and neutrophil activity highlights a mechanism that could be repurposed in cancer therapy to modulate tumor-associated inflammation and potentially suppress cancer progression. In addition, pathway-focused cellular studies have described modulation of MAPK/ERK [39] and PI3K/Akt signaling [40], attenuation of NF-κB activation [41], and regulation of oxidative stress [42] and mitochondrial activity [43] in a context-dependent manner. This suggests that imperatorin exerts a multifaceted impact on several signaling pathways.

**Figure 2:**
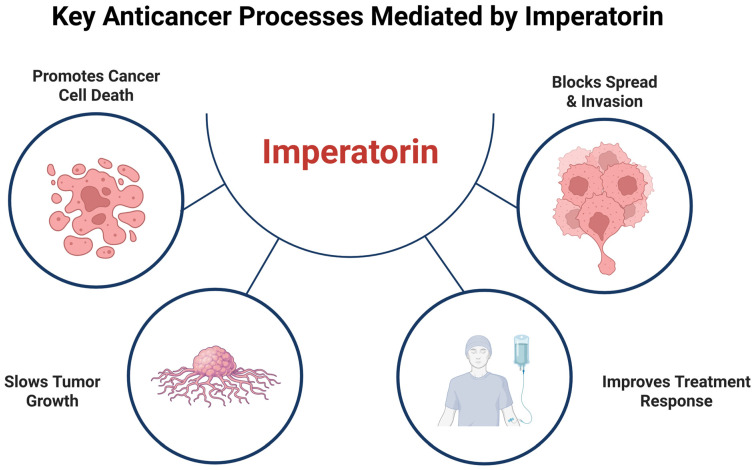
Conceptual overview of key anticancer processes mediated by imperatorin. This schematic summarizes the principal biological effects attributed to imperatorin in experimental cancer models. The figure serves as a guiding framework for the review and does not represent a definitive mechanistic model. Each process illustrated will be critically examined in subsequent sections based on available experimental evidence. Created in BioRender (https://biorender.com/a0jcieu), last accessed on 16 December 2025.

However, despite these promising findings, the research on imperatorin’s anticancer properties remains fragmented: studies vary widely in their experimental models, concentrations used, and outcome measures; many are limited to in vitro systems, and essential aspects such as pharmacokinetics, bioavailability, toxicity, and possible synergistic effects with other drugs remain underexplored. To provide an overarching framework for the discussion that follows, Figure 2 presents a conceptual overview of the principal anticancer processes attributed to imperatorin in experimental studies to date. Rather than offering a definitive mechanistic model, this schematic summarizes the significant biological effects reported across the literature. This figure is intended to orient the reader and serve as a guiding framework for the subsequent sections, in which each proposed anticancer action of imperatorin will be examined in detail based on individual in vitro and in vivo studies. A comprehensive, integrative model synthesizing these findings will be presented at the conclusion of the review.

Although imperatorin has been discussed in previous reviews [31,44,45], those works primarily addressed it as a broadly bioactive phytochemical or focused on non-oncological contexts, with anticancer activity treated as a secondary aspect. In contrast, the present review adopts a cancer-centered perspective, positioning imperatorin explicitly as an emerging anticancer lead compound. Preclinical evidence is comprehensively organized by tumor type, enabling direct comparison of efficacy, molecular mechanisms, and biological relevance across diverse malignancies—an approach not previously applied to imperatorin.

This review provides an integrated mechanistic synthesis, highlighting how imperatorin modulates apoptotic signaling, oncogenic survival pathways, angiogenesis, inflammation, immune responsiveness, and drug resistance. Particular emphasis is placed on imperatorin’s ability to reverse drug resistance, enhance the efficacy of chemotherapy, radiotherapy, and immune-cell–mediated cytotoxicity, and disrupt tumor–microenvironment interactions. In addition, the review critically evaluates pharmacokinetic and translational limitations, including bioavailability, metabolism, and safety gaps, and discusses strategies to overcome these barriers.

By integrating recent experimental findings with a tumor-specific and translationally oriented analysis, this review offers a distinct and updated contribution to the literature that extends beyond prior pharmacological surveys and provides a focused framework for future oncological development of imperatorin.

In this context, the present article aims to provide a comprehensive review of imperatorin—summarizing its chemical nature and natural sources; critically examining in vitro and in vivo studies on its anticancer effects; elucidating underlying molecular mechanisms; discussing pharmacokinetic behavior, bioavailability, and safety profiles; and identifying current challenges and future research directions. By integrating scattered evidence into a cohesive framework, we aim to assess whether imperatorin holds real promise as a lead compound or adjunct therapy in cancer prevention or treatment. Through this synthesis, we hope to inform and stimulate further research into imperatorin’s translational prospects, ultimately contributing to the ongoing search for novel, effective, and safer anticancer agents.

## 2. Imperatorin: Unveiling Its Biosynthesis, Physicochemical Properties, and Pharmacokinetics

Imperatorin is a linear furanocoumarin found mainly in *Apiaceae* and *Rutaceae* plants, and its extraction requires methods that take into account both its low polarity and the complexity of plant matrices, including factors like the stability of the compound, the relative matrix concentration, and the properties of the co-metabolites. The process usually begins with liquid–solid extraction using solvents such as petroleum ether, dichloromethane, ethyl acetate, methanol, or ethanol. Although traditional Soxhlet extraction is available, it is often less suitable because the high temperature can cause thermal degradation of furanocoumarins, resulting in comparatively low yields. More efficient modern techniques include ultrasound-assisted extraction, microwave-assisted extraction, ASE, and SFE [31,46,47].

Among these technologies, ASE consistently provides some of the highest yields, particularly for hydrophobic furanocoumarins. In *Archangelica officinalis* Hoffm. fruits, extraction with petroleum ether, followed by methanol at 100 °C and 60 bar, yielded about 19 mg/g of imperatorin [48]. Similarly, ASE yielded over 15 mg/g from *Pastinaca sativa* fruits [49]. Temperature is also vital for *Heracleum leskowii*, where higher temperatures gradually increased the recovery of imperatorin [47]. Supercritical CO_2_ extraction offers both high selectivity and low impurity levels. Under optimized conditions, SFE has yielded highly enriched extracts from sources such as *Citrus maxima*, yielding roughly 1.3 mg/g of imperatorin [50,51].

Because crude plant extracts contain numerous structurally related metabolites, further purification is necessary. Column chromatography is typically one of the first purification steps [52]. Depending on the extract, column purification can yield small but concentrated amounts of imperatorin; for example, silica-gel column chromatography yielded about 0.35 mg from 1 g of *A. dahurica* herb material [53]. Preparative thin-layer chromatography is often used after column chromatography to refine separation when coumarins co-elute. Counter-current chromatography provides a highly efficient liquid–liquid alternative that avoids irreversible adsorption to solid phases and often yields material of very high purity, usually around 98–100% [31]. Depending on the plant and extraction load, counter-current chromatography can produce significant quantities of purified compound, such as nearly 30 mg from *A. dahurica* herb or more than 100 mg from *Cnidium monnieri* fruits [54,55].

For quality control and quantitative determination, imperatorin is analyzed using HPLC–UV, GC–MS, and LC–MS. LC–MS offers high sensitivity and specificity. However, other methods are being tested and developed, including HPLC–ESI–MS coupled with HF–LPME to improve the determination between imperatorin and its metabolites [56].

Taken together, the data show considerable variation in imperatorin content across plant species. Some of the highest concentrations are found in the fruits of *A. officinalis*, which may contain about 19 mg/g, and in *P. sativa* fruits, where yields of around 15 mg/g are typical. *C. maxima* peel contains around 1 mg/g [51]. These differences underscore the importance of carefully selecting both the plant source and the extraction method when isolating imperatorin for analytical or biological studies.

In addition to imperatorin, the extraction of *Apiaceae* and *Rutaceae* plant matrices frequently yields structurally related furanocoumarins, including isoimperatorin and phellopterin [57,58]. Notably, isoimperatorin has been reported to exhibit anticancer activity by inhibiting tumor cell proliferation and inducing apoptosis through mitochondrial-dependent mechanisms, including cell cycle arrest and modulation of pro-apoptotic signaling pathways [59]. Phellopterin has been reported to exhibit anticancer activity by suppressing cancer cell proliferation, inducing cell cycle arrest and apoptosis, and attenuating chemoresistance by modulating oncogenic signaling pathways [60]. Therefore, the potential contribution of these co-extracted furanocoumarins to the overall anticancer activity of imperatorin-rich extracts should be considered when interpreting biological outcomes.

A summary of the reported plant sources of imperatorin, together with the extraction methods and corresponding contents or yields, is provided in Table 1.

### 2.1. Biosynthesis of Imperatorin: Pathways and Regulatory Mechanisms

The biosynthesis of imperatorin in plants proceeds via the general coumarin/furanocoumarin biosynthetic routes, beginning from the shikimate/phenylpropanoid pathway and followed by modifications unique to furanocoumarins. As with other coumarins, the path originates from the amino acid phenylalanine. This is converted through canonical enzymes of the phenylpropanoid pathway—notably PAL, C4H, and 4CL—leading to the formation of a precursor for coumarin biosynthesis [61].

Subsequent enzymatic steps yield simple coumarins (e.g., via hydroxylations). For furanocoumarins like imperatorin, the pathway diverges: a coumarin core (e.g., umbelliferone) undergoes prenylation (attachment of a prenyl group) that serves as a structure for producing the characteristic furanocoumarin structure [62].

In the case of the medicinal plant *A. dahurica* (a significant source of imperatorin), recent multi-omics studies identified that particular cytochrome-P450 enzymes mediate the hydroxylation of the psoralen backbone at specific positions: CYP71AZ18 catalyzes hydroxylation at the C-5 position to yield bergaptol, whereas either CYP71AZ19 or CYP83F95 acts at C-8 to produce xanthotoxol. These represent critical branching points in the furanocoumarin biosynthesis leading to compounds such as imperatorin (from bergaptol) and related derivatives [63].

The prenylation (addition of a prenyl group) is a decisive modification for imperatorin’s structure. In a study of a related species (or variety) of *A. dahurica*, a gene named AdOPT1 was shown in vitro to catalyze the oxygen-prenylation reaction between DMAPP and bergaptol—yielding isoimperatorin (the isomeric prenylated furanocoumarin) rather than imperatorin itself [64]. These findings demonstrate that prenylation is enzyme-mediated by a PT; however, the specific PT responsible for imperatorin formation has not yet been completely functionally characterized.

Recent genomic and transcriptomic work on *A. dahurica* has shed light on regulatory aspects controlling furanocoumarin production (including imperatorin). Gene expression modules linked to coumarin/furanocoumarin content and transcriptome analyses identified clusters (modules) of co-expressed genes associated with coumarin and terpenoid backbone biosynthesis. Specifically, specific modules comprising cytochrome P450s (e.g., CYPs), PTs, UGTs, and other enzymes correlated with the levels of imperatorin, isoimperatorin, and related furanocoumarins [65].

In this context, epigenetic/chromatin-level regulation in *A. dahurica* may involve ACRs, particularly proximal ACRs near biosynthetic genes, which are correlated with high expression of the key CYP genes (CYP71AZ18, CYP71AZ19, CYP83F95) involved in furanocoumarin biosynthesis. This suggests that chromatin accessibility plays a role in regulating metabolite biosynthesis levels [63]. Quantification of coumarin content across root developmental stages showed that furanocoumarin levels, including imperatorin, are modified as roots mature [66]. Environmental/stress-induced regulation may also play a role. Although this phenomenon has not yet been fully characterized for imperatorin specifically, general knowledge of coumarin biosynthesis indicates that both biotic and abiotic stresses can influence the expression of enzymes involved in coumarin biosynthesis, thereby modulating levels of secondary metabolites [61].

In summary, the biosynthesis of imperatorin appears to be part of the broader furanocoumarin pathway in plants, originating from phenylalanine via the phenylpropanoid pathway, leading to a coumarin core (e.g., psoralen/bergaptol), which is then prenylated and further modified. Recent advances have identified key enzymes (especially cytochrome P450 hydroxylases) and regulatory mechanisms (transcriptional modules, chromatin accessibility, developmental timing) in a model source plant.

However, important gaps remain in our understanding of imperatorin biosynthesis. Although PTs involved in imperatorin formation have been reported, the enzymatic steps underlying regioselective oxygen-prenylation and their regulation are not yet fully resolved. This incomplete knowledge limits a comprehensive understanding of imperatorin biosynthesis. In addition, downstream modifications (e.g., further tailoring reactions, glycosylation, storage, transport) and their regulatory mechanisms remain poorly characterized. Given the pharmacological interest in imperatorin, filling these gaps through gene discovery, functional assays, and regulation studies represent a promising direction for future research.

While total chemical syntheses specifically targeting imperatorin are not frequently reported as standalone routes in the classic synthetic literature, there are well-established strategies for constructing the furocoumarin scaffold to which imperatorin belongs. Reviews on furocoumarin synthesis describe methods that form the furan ring onto a coumarin core as a general approach to linear furocoumarins, as well as contemporary advances in the synthesis of coumarin–furan heterocycles, which are broadly relevant to this class of compounds [67,68]. In addition, chemists have reported the synthesis of imperatorin derivatives and related furocoumarin analogs using substrates such as xanthotoxin or other coumarin scaffolds, demonstrating practical synthetic routes for modifying and accessing structural motifs closely related to imperatorin [69,70]. These studies indicate that while extraction remains the dominant method for obtaining imperatorin for most applications, organic synthetic approaches to furocoumarin frameworks exist and have been applied to both analog design and broader synthetic efforts.

### 2.2. Physicochemical Properties of Imperatorin: Structural Characteristics and Stability

Imperatorin is a furanocoumarin derivative (a coumarin subclass) with molecular formula C_16_H_14_O_4_. In its pure form, it typically appears as white crystals or as long, fine needles. As for solubility, imperatorin is essentially insoluble in water under standard conditions, but is soluble in some non-polar solvents [31].

According to PubChem from the National Library of Medicine (USA) (https://pubchem.ncbi.nlm.nih.gov/compound/Imperatorin, last accessed in December 2025), imperatorin’s molecular weight is 270.28 g/mol, reflecting a composition of 20 heavy atoms and no formal charge. The molecule contains no hydrogen bond donors and four hydrogen bond acceptors. Its calculated XLogP3 value is 3.4, and, structurally, imperatorin has three rotatable bonds and a complexity value of 436. Still, it lacks any defined or undefined stereocenters and contains only one covalently bonded unit. Together, these properties outline imperatorin as a moderately lipophilic, structurally rigid molecule with well-defined physicochemical characteristics relevant to its biological behavior. Experimentally, imperatorin is described as a solid with a crystalline appearance, forming prisms from ether, long needles from hot water, or crystals from alcohol, depending on the solvent system. It melts at 102 °C. Its solubility behavior aligns with its computed lipophilicity: the compound is only slightly soluble in water and remains very sparingly soluble even in boiling water, but it dissolves in organic solvents such as chloroform, benzene, ether, ethanol, and petroleum ether. It is also soluble in alkali hydroxide solutions. Under typical storage conditions, imperatorin is stable, though like many organic compounds, it decomposes under fire, producing carbon oxides. When heated to decomposition, it can release acrid smoke and fumes. Taken together, these properties are consistent with imperatorin’s characterization as an organic solid with well-defined chromatographic behavior.

### 2.3. Pharmacokinetics of Imperatorin: Evaluating the Phytochemical’s Absorption, Distribution, Metabolism, and Toxicity

The oral bioavailability of imperatorin appears relatively low under standard conditions. In rat models, the absolute oral bioavailability ranged from approximately 3.9% to 34.8%, depending on dose, suggesting limited absorption and/or extensive first-pass metabolism [71]. Additional studies using Caco-2 cell models indicate that imperatorin can influence the intestinal uptake of co-administered compounds, possibly by modulating cell membrane potential, inhibiting P-gp efflux, and loosening tight junction proteins to promote paracellular transport. This behavior suggests that imperatorin may enhance drug absorption beyond its own uptake [72].

Imperatorin demonstrates high plasma protein binding—above 90% in rat plasma—indicating that a significant portion of the circulating compound is bound to proteins, which may slow its distribution into tissues [71]. Studies in animal models (mice) have further shown that imperatorin and related coumarins exhibit good permeability across biological barriers, including the blood–brain barrier, suggesting potential distribution in the central nervous system [73]. In formulation contexts such as multi-component botanical extracts, imperatorin may also influence the pharmacokinetics of other compounds, modulating their plasma concentrations [74].

Metabolic processing is an essential determinant of imperatorin’s pharmacokinetics. In vitro studies using liver microsomes have identified demethylation and oxygenization as principal metabolic pathways, producing metabolites such as xanthotoxol and heraclenin [71]. These phase I transformations suggest involvement of cytochrome P450 enzymes. Indeed, imperatorin has been shown to interact with human CYP450 isoforms, such as CYP2A6 and CYP2A13, and can act as a time-dependent inhibitor [75]. Furthermore, research indicates that imperatorin (and its structural analogs) can inhibit several CYP450 enzymes, including CYP1A2, CYP2B6, and others, illuminating potential causes of drug–drug interactions [76]. For example, co-administration with diazepam in rats demonstrated that imperatorin can inhibit diazepam’s metabolism, increasing its systemic exposure (AUC and Cmax) while reducing clearance, underlining clinical considerations for herb–drug interactions [77].

While imperatorin exhibits a range of biological activities with therapeutic promise, toxicological data are limited but notable. Some in silico ADMET analyses suggest potential toxicity patterns, though imperatorin shows a high predicted intestinal absorption in such models [78]. Empirical toxicological evaluations remain sparse, and existing reviews point out that side effects have been observed in some studies, yet detailed in vivo toxicity profiles are lacking and require further research [44]. Significantly, the inhibitory effects on CYP450 enzymes raise concerns that imperatorin may alter the metabolism and toxicity profiles of co-administered drugs, further complicating its safety assessment [75].

## 3. Anti-Inflammatory and Antioxidant Pharmacodynamics of Imperatorin: Mechanisms of Action and Therapeutic Potential

Imperatorin has gained growing research attention for its broad spectrum of biological activities. Among these, its anti-inflammatory and antioxidant properties have been extensively characterized, positioning the compound as a promising candidate in the development of phytotherapeutics for inflammation- and oxidative stress-related diseases.

The anti-inflammatory pharmacodynamics of imperatorin involve coordinated regulation of multiple intracellular signaling pathways that contribute to both the initiation and amplification of inflammatory responses. Experimental studies consistently report that imperatorin suppresses the expression of COX-2 and iNOS, thereby reducing the production of prostaglandins and nitric oxide—two key mediators involved in acute and chronic inflammation [79,80,81]. This enzymatic suppression is closely associated with inhibition of the NF-κB signaling pathway, as imperatorin interferes with the phosphorylation and degradation of IκBα, ultimately preventing nuclear translocation of NF-κB and downregulating its downstream pro-inflammatory cytokines such as TNF-α, IL-1β, and IL-6 [82,83]. In addition to NF-κB modulation, imperatorin has been shown to inhibit JAK/STAT and MAPK pathways, including ERK, JNK, and p38 MAPK, thereby dampening cytokine signaling and inflammatory gene transcription [41,80]. Further mechanistic insight has emerged from evidence showing that imperatorin modulates the PI3K/Akt/NF-κB axis, which integrates signals involved in cellular survival, immune activation, and inflammation. By attenuating the phosphorylation of PI3K, Akt, and p65, imperatorin disrupts critical upstream activators of NF-κB, thereby reinforcing its anti-inflammatory efficacy [40,84]. This multi-target regulation highlights imperatorin’s broad capacity to intervene in the inflammatory cascade at several hierarchical levels.

**Figure 3:**
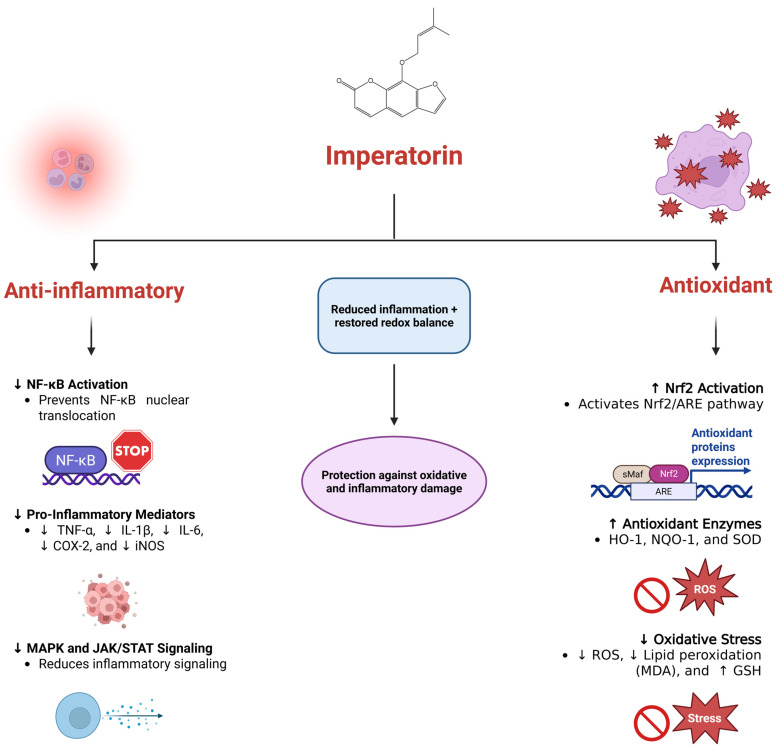
Dual anti-inflammatory and antioxidant mechanisms of imperatorin. The figure illustrates the dual biological actions of imperatorin, a furanocoumarin compound, highlighting its capacity to reduce inflammation and oxidative stress. On the anti-inflammatory side (**left**), imperatorin inhibits NF-κB activation by preventing its nuclear translocation, decreases pro-inflammatory mediators (including TNF-α, IL-1β, IL-6, COX-2, and iNOS), and reduces MAPK and JAK/STAT signaling. On the antioxidant side (**right**), imperatorin activates the Nrf2/ARE pathway, increases the expression of antioxidant enzymes (HO-1, NQO-1, and SOD) and lowers oxidative stress by reducing ROS and lipid peroxidation while increasing GSH. Together, these actions restore redox balance and protect against oxidative and inflammatory damage. Created in BioRender (https://biorender.com/rgcypml), last accessed on 16 December 2025.

In parallel with its anti-inflammatory actions, imperatorin exhibits notable antioxidant pharmacological effects, primarily by activating endogenous cytoprotective mechanisms. A central component of this antioxidant capacity involves stimulation of the Nrf2/ARE pathway, which promotes nuclear translocation of Nrf2 and subsequent upregulation of antioxidant enzymes such as HO-1 and NQO-1. Activation of these enzymes contributes to the detoxification of ROS, restoration of redox balance, and protection of cellular structures from oxidative injury [30,42,85,86]. Imperatorin has also been shown to enhance SOD activity and GSH levels while lowering markers of lipid peroxidation, such as MDA [43,87]. The combined antioxidant effects provide significant protection against oxidative stress. Figure 3 illustrates the dual anti-inflammatory and antioxidant mechanisms of imperatorin, as reported in the studies included in this section.

The compound’s combined anti-inflammatory and antioxidant effects contribute to its therapeutic potential in numerous pathological contexts. Preclinical studies suggest benefits in models of atherosclerosis, where imperatorin suppresses vascular inflammation and protects endothelial integrity [88]; in neurodegenerative conditions, where it mitigates oxidative injury and neuroinflammation [89]; and in metabolic disorders, where it modulates inflammatory signaling associated with insulin resistance and lipid metabolism [90]. Additional reports have shown protective effects against ischemia–reperfusion injury [91], pulmonary inflammation [80], and endometriosis [84], indicating broad pharmacological applicability. These findings support the potential utility of imperatorin in diseases characterized by chronic low-grade inflammation and oxidative imbalance. Further clinical studies, along with refined pharmacokinetic and toxicological profiling, are required to establish optimal dosing, long-term safety, and the feasibility of incorporating imperatorin into therapeutic regimens, either as a standalone agent or in combination with existing anti-inflammatory or antioxidant therapies.

Given that chronic inflammation and oxidative stress are fundamental drivers of tumor initiation and progression, the molecular pathways targeted by imperatorin—particularly NF-κB, JAK/STAT, MAPK, PI3K/Akt, and Nrf2—are also central to cancer biology [92,93]. The compound’s ability to suppress pro-inflammatory signaling while enhancing antioxidant defenses suggests potential relevance in modulating the tumor microenvironment and interrupting oncogenic processes, thereby positioning imperatorin as a candidate of interest for cancer-related research. 

## 4. Imperatorin in Cancer Prevention and Intervention

This section examines the expanding research landscape on imperatorin’s anticancer properties. Emerging evidence indicates that imperatorin can influence several fundamental processes involved in cancer development, including uncontrolled cell proliferation, programmed cell death, and the spread of malignant cells. Increasingly, studies suggest that imperatorin acts on diverse molecular targets, positioning it as a multifaceted compound with therapeutic promise. This review synthesizes current insights into the mechanisms by which imperatorin exerts its anticancer effects, assesses its potential clinical value, and discusses how it may advance future cancer treatment strategies.

### 4.1. Literature Search Report

**Figure 4:**
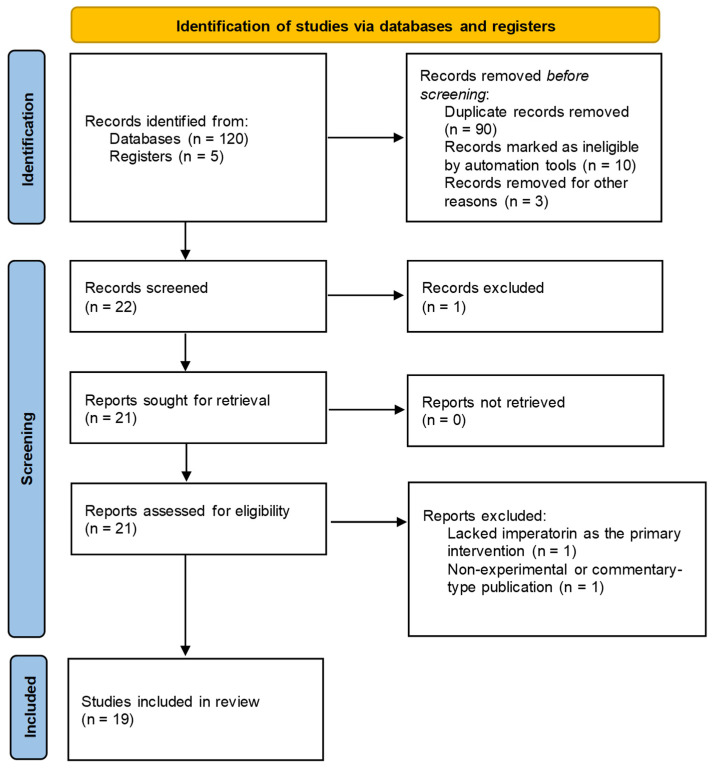
PRISMA flow chart depicting the study identification, screening, and selection process. Based on Page et al. [94].

A comprehensive description of the review methodology is provided in Appendix A, where full procedural details and supporting information are presented. A total of 125 records were identified during the initial search phase, including 120 from database searches and 5 from supplementary sources and registers. Before screening, 103 records were removed: 90 duplicates, 10 entries automatically flagged as ineligible, and 3 records excluded for other technical reasons, leaving 22 eligible for screening. Following title and abstract screening, one record was excluded, leaving 21 reports for full-text retrieval. All reports were successfully obtained (0 not retrieved) and assessed for eligibility. Of these, two reports were excluded based on predefined criteria: one lacked imperatorin as the primary experimental intervention, and another was a non-experimental or commentary-type publication. Ultimately, 19 studies met all inclusion criteria and were incorporated into the final qualitative synthesis. The complete selection process and filtering stages are illustrated in Figure 4, which presents the PRISMA flow diagram summarizing identification, screening, eligibility assessment, and inclusion. Table 2 summarizes the included studies in this review.

### 4.2. Preclinical Anticancer Studies of Imperatorin: Mechanisms, Efficacy, and Potential Clinical Implications

This section provides a comparative overview of cancer research on imperatorin. Table 2 summarizes investigations reporting the anticancer potential of imperatorin. To support a more straightforward interpretation of how imperatorin may function across diverse biological settings, the subsequent subsections are arranged by cancer type. This structure helps identify potential differences in activity, mechanisms, or therapeutic relevance across tumor contexts. By examining findings within individual cancer categories, patterns can be more easily recognized, and consistency across studies can be assessed.

#### 4.2.1. Liver Cancer

Liver cancer—primarily HCC—arises from chronic hepatic inflammation leading to fibrosis, cirrhosis, and genetic/epigenetic alterations in hepatocytes. Standard drivers include HBV/HCV infection, aflatoxin exposure, NASH, and alcohol [109]. Globally, liver cancer has a high incidence in Asia and sub-Saharan Africa due to viral hepatitis prevalence [110]. Treatment limitations include late-stage diagnosis [111], underlying liver dysfunction restricting therapy tolerance [112], high tumor heterogeneity [113], and limited response to systemic therapies despite advances in immunotherapy [114]. Recurrence rates remain high after resection or ablation [115].

The in vitro study conducted by Hu et al. [95] used HepG2, Hep3B, PLC, and Huh7 liver cancer cell lines treated with imperatorin at 2.5–80 μM for 24 or 48 h. The compound increased cisplatin cytotoxicity and reversed drug resistance in cisplatin-resistant HepG2 and Huh7 cells. When combined with cisplatin, imperatorin produced strong synergistic effects, enhancing apoptosis and inducing mitochondrial membrane potential collapse more effectively than either agent alone. Mechanistically, these effects were associated with suppression of MCL-1 expression.

Using HepG2, Hep3B, and multidrug-resistant HepG2 cells in vitro, along with a multidrug-resistant HepG2 xenograft nude mouse model in vivo, Li et al. [96] evaluated imperatorin at IC_50_ values of 43.3 μM (HepG2) and 28.1 μM (resistant HepG2) and concentrations of 40–200 μM for 48 h. In vivo, mice received 50 mg/kg intravenously every two days for 14 days. Imperatorin suppressed multidrug-resistant HepG2 cell growth and enhanced apoptosis in vitro, while inhibiting tumor growth in vivo. Mechanistic analyses showed proteasomal degradation of MCL-1, increased Fas receptor expression, Bak activation, Bax mitochondrial translocation, caspase-3/8/9 activation, cytochrome c release, and mitochondrial membrane potential disruption in vitro; MCL-1 degradation was also observed in vivo.

The study by Luo et al. [35] examined imperatorin in HepG2 cells and in a nude mouse xenograft model. Reported IC_50_ values were 101.2 μM at 24 h, 60.5 μM at 48 h, and 22.4 μM at 72 h. In vivo, mice received 50 or 100 mg/kg orally for 14 consecutive days. Imperatorin reduced cell proliferation and increased apoptosis in vitro, while inhibiting tumor growth in vivo. Mechanistic findings included activation of caspases, PARP cleavage, cytochrome c release, mitochondrial membrane potential disruption, increased Fas receptor expression, upregulation of p21 and p53, and modulation of Bcl-2 family proteins (↓ Bcl-2; ↑ Bax, Bad, tBid). The in vivo mechanisms were not fully described.

Across the three studies, imperatorin consistently demonstrates anticancer activity against liver cancer models, both in vitro and in vivo. The compound enhances apoptosis, reduces proliferation, and disrupts mitochondrial membrane potential, often acting by modulating Bcl-2 family proteins, activating caspases, promoting cytochrome c release, and downregulating MCL-1 [35,95,96]. Two studies show that imperatorin can overcome or reverse drug resistance in multidrug-resistant and cisplatin-resistant liver cancer cells, synergizing particularly well with cisplatin [95,96]. In vivo xenograft models further confirm its ability to inhibit tumor growth at administered doses [35,96]. Overall, the studies collectively indicate that imperatorin exerts its anticancer effects primarily by triggering apoptotic pathways and destabilizing mitochondrial integrity, while also promoting proteasomal degradation of anti-apoptotic proteins.

#### 4.2.2. Lung Cancer

Lung cancer includes NSCLC and SCLC, both driven by mutations from carcinogen exposure—most commonly cigarette smoke—leading to uncontrolled proliferation and impaired apoptosis [116,117]. It remains the leading cause of cancer death worldwide. Although targeted therapies and immunotherapy have improved survival in selected patients, many tumors lack actionable mutations, resistance rapidly develops, and metastatic disease is often present at diagnosis [118,119,120,121]. Screening uptake with low-dose computed tomography also remains insufficient [122].

You et al. [97] study employed CD133^+^ and CD133^−^ A549 and PC9 lung cancer cells in vitro, as well as a CD133^+^ A549 xenograft model in BALB/c nude mice. Imperatorin at 10 μM for 12 h increased the sensitivity of CD133^+^ cancer cells to γδ T cell-mediated cytotoxicity and synergized with γδ T cell therapy to induce mitochondrial apoptosis. In vivo, mice received 50 mg/kg intraperitoneally twice weekly for 28 days, resulting in reduced tumor size, with the drug–cell therapy combination producing pronounced tumor suppression. Mechanistically, imperatorin downregulated MCL-1 expression in vitro and inhibited MCL-1 expression in vivo.

Using H1975 and A549 lung cancer cell lines in vitro and xenograft mouse models generated with H1975 (BALB/c *nu/nu*) and LLC cells (C57BL6/J), Xu et al. [32] evaluated imperatorin with IC_50_ values of 9.64 ± 3.50 μM (24 h) and 5.28 ± 0.50 μM (48 h) for H1975 cells, and 18.20 ± 1.35 μM (24 h) and 14.17 ± 3.02 μM (48 h) for A549 cells. In vivo dosing was 20 or 40 mg/kg intraperitoneally for 21 days. Imperatorin inhibited cell growth in vitro and reduced tumor size, volume, and weight in vivo. The compound modulated the PI3K/Akt and PD-L1 pathways in vitro and, in vivo, suppressed PI3K/Akt signaling and downregulated PD-L1 expression in tumor tissues.

The study by Choochuay et al. [98] used NCI-H23, NCI-H292, and A549 lung cancer cell lines, treating them with 0.1–10 μg/mL imperatorin for 12 or 24 h. The treatment increased apoptosis after detachment, suppressed anchorage-independent growth, and sensitized cells to anoikis. Mechanistic observations included increased p53 protein levels, downregulation of MCL-1, and upregulation of Bax, indicating activation of mitochondrial apoptotic pathways.

Across studies investigating lung cancer models, imperatorin consistently demonstrates pro-apoptotic and antitumor effects in vitro and in vivo, acting by suppressing anti-apoptotic proteins, modulating immune sensitivity, and regulating survival signaling pathways. In CD133^+^ cells, imperatorin enhances γδ T cell-mediated cytotoxicity and mitochondrial apoptosis while reducing MCL-1 expression, with combined γδ T cell therapy producing strong tumor suppression in xenograft models [97]. Additional work in H1975, A549, and LLC tumor models shows growth inhibition and tumor reduction associated with modulation of the PI3K/Akt and PD-L1 pathways [32]. Imperatorin also increases anoikis sensitivity, suppresses anchorage-independent growth, and regulates p53, Bax, and MCL-1 in multiple lung cancer cell lines, including NCI-H23, NCI-H292, and A549 [98]. Collectively, these findings indicate that imperatorin impairs cell survival and enhances apoptotic signaling across diverse lung cancer systems.

#### 4.2.3. Glioblastoma

Glioblastoma originates from glial cells and is characterized by aggressive infiltration, extensive necrosis, and high genetic instability [123,124,125]. Alterations in EGFR, PTEN, and MGMT methylation status drive progression [126]. Glioblastoma carries a poor prognosis [127]. Surgical resection is limited by diffuse invasion into healthy brain tissue, and the blood–brain barrier restricts effective drug delivery [128,129]. Radiotherapy and temozolomide offer modest survival benefits, and resistance develops quickly [130,131]. Immunotherapy has not yet shown consistent success [132].

In glioblastoma models, imperatorin demonstrates apparent pro-apoptotic activity, enhancing programmed cell death in T98G cells and acting synergistically with quercetin to further increase apoptosis [99]. These effects are associated with the reduction of heat-shock proteins HSP27 and HSP72 and increased activation of caspases 3 and 9, indicating that imperatorin acts through mitochondrial apoptotic pathways. This in vitro study used the T98G glioblastoma cell line treated with imperatorin at 25, 50, and 100 µM for 24 or 48 h.

#### 4.2.4. Cervical Cancer

Cervical cancer develops through persistent infection with high-risk HPV, leading to dysplasia and malignant transformation in cervical epithelial cells [133]. It is more prevalent in low- and middle-income countries [134]. While surgery, chemoradiation, and targeted immunotherapy (e.g., PD-1 inhibitors) can be effective, treatment barriers include late presentation, limited screening resources, and resistance in recurrent or metastatic disease [135,136,137,138]. Prevention remains the most critical aspect of nations’ efforts against cervical cancer [139].

The in vitro study by Wang et al. [40] used HeLa cervical cancer cells treated with imperatorin at 5–150 µM for 8, 12, or 24 h. Imperatorin produced both pro-apoptotic and anti-inflammatory effects. Mechanistically, it inhibited TNF-α-induced expression of NF-κB target genes and suppressed NF-κB activation by blocking TNF-α-stimulated phosphorylation of IKKα/β and IκB, preventing IκB degradation and NF-κB p65 nuclear translocation. The compound also reduced TNF-α-induced PI3K/Akt activation and decreased ROS production, indicating broad interference with inflammatory and survival signaling pathways. Treatment inhibits TNF-α-induced transcription of NF-κB target genes, blocks upstream phosphorylation events required for NF-κB activation, and reduces ROS generation, collectively contributing to its apoptotic and anti-inflammatory effects.

#### 4.2.5. Breast Cancer

Breast cancer arises through hormonal, genetic (e.g., BRCA1/2), and environmental factors that drive mutations in mammary epithelial cells. It is the most common cancer in women globally. Although early detection and subtype-specific treatments (endocrine therapy, HER2-targeted therapy, chemotherapy) have improved survival, limitations include treatment resistance, heterogeneity within tumors, and the lack of effective targeted options for triple-negative breast cancer [140,141,142,143,144,145]. Long-term toxicities and access disparities also hinder outcomes [146,147].

The in vitro study by Amini et al. [100] used the MCF-7 breast cancer cell line exposed to imperatorin at 0.1–1.5 µM for 24 h. Imperatorin decreased cell viability and showed synergistic effects with radiotherapy or hyperthermia, jointly suppressing proliferation and promoting apoptosis. Mechanistic analyses revealed increased expression of Bax and activation of caspases 3, 8, and 9, along with downregulation of Bcl-2, indicating induction of both intrinsic and extrinsic apoptotic signaling pathways. These shifts in Bax/Bcl-2 balance and caspase activation support a transition toward mitochondrial-mediated apoptosis.

#### 4.2.6. Esophageal Cancer

Esophageal cancer includes squamous cell carcinoma (linked to tobacco, alcohol, and chronic irritation) and adenocarcinoma (associated with Barrett’s esophagus and gastroesophageal reflux disease) [148]. Incidence patterns vary: squamous cell carcinoma dominates in Asia and Africa, while adenocarcinoma is increasing in Western countries [149,150]. Treatment often involves chemoradiation and surgery, but the detection patterns of early-stage esophageal cancer are low; therefore, most patients are first diagnosed when the cancer has metastasized [151,152]. Because many patients present at advanced stages, the benefit of starting systemic therapy must be carefully evaluated [153]. High recurrence rates persist even after aggressive multimodal treatment [154].

Xu et al. [101] evaluated imperatorin in vitro using KYSE30 and KYSE150 esophageal cancer cells and in vivo using two xenograft models: luciferase-expressing KYSE150 cells in nude mice and luciferase-expressing EC9706 cells in NCG mice. Cells were treated with 40 or 80 µM imperatorin for 24 h, resulting in reduced invasive potential and decreased angiogenic activity. In vivo, oral administration of 25 or 50 mg/kg two to three times per week inhibited metastasis to the lungs, liver, kidneys, and spleen and reduced tumor angiogenesis. Mechanistically, imperatorin decreased TGF-β2 expression, suppressed CREB1 transcriptional activity, inhibited ERK signaling, reduced CAF-secreted CCL2, upregulated E-cadherin, and downregulated fibronectin, N-cadherin, MMP2, and MMP9 in vitro; in vivo, it consistently inhibited CREB1 activity, TGF-β2-ERK signaling, and fibroblast-derived CCL2. Overall, the findings indicate that imperatorin effectively disrupts pro-metastatic signaling in esophageal cancer.

#### 4.2.7. Gastric Cancer

Gastric cancer typically begins with chronic inflammation from *Helicobacter pylori* infection, progressing to atrophy, dysplasia, and carcinoma [155]. This type of cancer is more common in East Asia, which corresponds to an area of high *H. pylori* prevalence [156]. Although surgery is the cornerstone of early disease management, most cases are detected late because symptoms often onset late [157,158]. Immunotherapy offers incremental benefits but is limited by tumor heterogeneity and resistance [159]. Screening programs (e.g., endoscopy and imaging techniques), although effective, are usually invasive, high-cost, and resource-consuming [160].

The in vitro study by Am et al. [102] used SGC-7901 gastric cancer cells treated with imperatorin at an IC_50_ of 62.6 µM. Treatment inhibited cell growth, increased apoptosis, promoted DNA damage, caused cell shrinkage, and distorted typical cellular structures. Mechanistically, imperatorin downregulated PI3K/Akt/mTOR signaling proteins and induced sub-G1 cell cycle arrest, indicating activation of apoptosis and disruption of proliferative signaling. These results suggest that imperatorin effectively triggers multiple mechanisms leading to gastric cancer cell death.

#### 4.2.8. Colon Cancer

Colon cancer develops through the adenoma–carcinoma sequence driven by mutations in APC, KRAS, and p53, often influenced by diet, lifestyle, and genetics [161,162]. It is increasing among younger adults in high-income countries [163]. Screening colonoscopy improves survival by detecting precancerous lesions. However, treatment limitations include drug resistance, limited benefit from immunotherapy in microsatellite-stable tumors, and metastatic disease at presentation in a significant subset [164,165,166,167]. Access to screening and early intervention remains uneven for many minority groups [168].

The in vitro study by Zheng et al. [34] used HT-29 colon cancer cells treated with imperatorin at IC_50_ values of 239 µM (24 h), 101 µM (48 h), and 78 µM (72 h). The compound inhibited cell proliferation and viability while increasing apoptosis. Mechanistically, imperatorin upregulated p53, activated caspase-3 and caspase-7, increased the Bax/Bcl-2 ratio, induced G1 cell cycle arrest, and elevated ROS levels, indicating activation of mitochondrial apoptotic pathways.

The study by Mi et al. [37] employed HCT116 cells in vitro and an HCT116 xenograft model in BALB/c nude mice. Cells were treated with 10–150 µM imperatorin for 12 or 24 h, resulting in inhibition of cell proliferation. In vivo, mice received oral doses of 50 or 100 mg/kg three times per week for 40 days, leading to reduced tumor growth and angiogenesis. Mechanistically, imperatorin suppressed HIF-1α protein synthesis and levels, downregulated VEGF and EPO mRNA, induced G1 cell cycle arrest, and inhibited mTOR/p70S6K/4E-BP1 and MAPK signaling both in vitro and in vivo, while reducing VEGF and CD31 expression in tumor tissues.

Imperatorin exhibits vigorous anticancer activity in colon cancer models, inhibiting proliferation and inducing apoptosis in vitro, and suppressing tumor growth and angiogenesis in vivo. Mechanistically, its effects are mediated by activation of apoptotic pathways, ROS generation, cell cycle arrest, and downregulation of hypoxia- and growth-signaling pathways [34,37]. Overall, imperatorin effectively impairs colon cancer cell survival and tumor progression.

#### 4.2.9. Osteosarcoma

Osteosarcoma is a mesenchymal neoplasm and is characterized by genomic instability and production of immature osteoid. It is the most common primary bone cancer, affecting adolescents and young adults [169]. Although advancements in chemotherapy and surgical methods have enhanced survival rates, the results for high-risk cases continue to be less than ideal, especially when there are metastases, a poor histologic response to chemotherapy, or insufficient surgical margins [170]. Biological heterogeneity [171], early lung metastasis [172], and lack of effective targeted therapies limit progress [173]. Immunotherapy has shown limited benefit, and few new drugs have significantly changed outcomes in the past few decades [174].

The study by Lv et al. [103] used U2OS and 143B osteosarcoma cell lines in vitro and a 143B xenograft model in BALB/c nude mice. In vitro IC_50_ values were 131.4 μM (24 h) and 116.3 μM (48 h) for U2OS cells, and 118.7 μM (24 h) and 90 μM (48 h) for 143B cells. Imperatorin inhibited cell proliferation, migration, and invasion, and induced autophagy, as indicated by increased expression of ATG1, ATG5, and LC3B. The compound caused G0/G1 cell cycle arrest by downregulating cyclin D1 and CDK6, and upregulating PTEN and p21, while reducing phosphorylation of Akt and mTOR. In vivo, treatment with 5 mg/kg intraperitoneally every other day for five doses reduced tumor growth, accompanied by increased PTEN- and LC3-positive cells and p-Akt- and CDK6-negative cells in tumor tissues. Overall, imperatorin demonstrates potential as an anticancer agent by targeting both growth and survival pathways in osteosarcoma.

#### 4.2.10. Studies Evaluating Multiple Cancers

The in vitro study by Wu et al. [104] assessed imperatorin in KB-3-1, KB-V1, ovarian cancer (OVCAR-8, NCI-ADR-RES), lung cancer (H460, H460-MX20), and colon cancer (S1, S1-MI-80) cells. Imperatorin reversed ABCG2-mediated multidrug resistance, potentiated topotecan-induced apoptosis, and reduced cell viability. Mechanistically, it inhibited ABCG2-mediated drug efflux and increased intracellular accumulation of the ABCG2 substrate, pheophorbide A.

In vitro studies in leukemia (K562, doxorubicin-resistant K562) and ovarian cancer (A2780, taxol-resistant A2780) cells treated with 2.78–11.10 µM for 70–120 min showed that imperatorin restricted glycolysis and glutamine metabolism in resistant K562 cells, enhanced cytotoxicity of doxorubicin and taxol, and reversed drug resistance. Mechanistically, imperatorin increased intracellular Rho123 accumulation and decreased P-gp efflux activity [105].

Imperatorin was tested in vitro on liver cancer (SNU 449) and colon cancer (HCT-15) cells at 7.4–740 nmol/mL for 24 h and 4 days. The treatment inhibited cell growth and increased apoptosis, inducing G1–SubG1 cell cycle arrest and decreasing the Bcl-2/Bax ratio [106].

Grabarska et al. [107] used rhabdomyosarcoma (TE671), lung cancer (A549, H2170, H1299), and larynx cancer (RK33, RK45) cells treated with 1–200 µM imperatorin (IC_50_ values: TE671 111.2 µM, RK33 67.8 µM) for 24–72 h. The compound decreased cell viability and proliferation, and increased apoptosis, particularly in TE671 and RK33 cells. Mechanistically, imperatorin increased cleaved caspase-3, induced G1 cell cycle arrest, and modulated p21 and cyclin D1 expression.

The in vitro study by Bądziul et al. [108] examined cervical cancer (HeLa) and Hep-2 cells treated with 50 or 100 µM imperatorin for 24 or 48 h. Imperatorin reduced cell viability and promoted apoptosis, with synergistic enhancement when combined with quercetin. Mechanistically, the effects involved decreased HSP72 expression and increased caspase activity.

Across diverse cancer types, imperatorin exhibits broad-spectrum anticancer activity, including reversal of drug resistance, inhibition of proliferation, induction of apoptosis, and modulation of cell cycle and apoptotic signaling pathways. It enhances the cytotoxicity of chemotherapeutics such as topotecan, doxorubicin, and taxol and demonstrates synergistic effects with other compounds, such as quercetin [104,105,106,107,108]. Collectively, these studies highlight imperatorin’s potential as a versatile anticancer agent across multiple tumor models.

## 5. Conclusions

Imperatorin emerges from the current body of evidence as a promising natural compound with broad pharmacological relevance, particularly in cancer prevention and intervention. Its biological profile reflects a convergence of anti-inflammatory, antioxidant, pro-apoptotic, anti-proliferative, anti-angiogenic, and immunomodulatory effects, positioning it as a compelling phytochemical candidate for further oncological development. Across diverse experimental models, imperatorin consistently suppresses tumor cell viability, induces both intrinsic and extrinsic apoptotic pathways, and interferes with pivotal oncogenic signaling networks, including PI3K/Akt, MAPK, mTOR, NF-κB, and hypoxia-regulated pathways. Its ability to destabilize mitochondrial integrity, modulate Bcl-2 family proteins, promote caspase activation, and impair cell cycle progression underscores a mechanistic versatility that contributes to its anticancer potential.

Importantly, imperatorin does not act solely on tumor cells but also influences the tumor microenvironment, reducing inflammatory mediators, inhibiting angiogenesis, decreasing fibroblast-derived chemokines, and enhancing susceptibility to immune-cell-mediated cytotoxicity. These properties highlight its relevance not only for direct cytotoxicity but also for modifying the extrinsic factors that sustain tumor growth and metastatic progression. Preclinical studies reinforce these observations, demonstrating significant reductions in tumor volume, metastatic spread, and drug resistance across multiple cancer types.

Nonetheless, the therapeutic promise of imperatorin remains tempered by several limitations. Most studies rely on in vitro systems with variable dosing ranges, short exposure times, and inconsistent methodological designs, which complicate the interpretation and comparison of findings. Although in vivo data are encouraging, they remain limited in scope and rarely address long-term toxicity, pharmacodynamics, or clinically relevant dosing regimens. The compound’s low oral bioavailability, high plasma protein binding, and interactions with cytochrome P450 enzymes pose additional challenges for clinical translation. Moreover, although evidence suggests that imperatorin can reverse multidrug resistance and enhance the efficacy of standard chemotherapeutics, the molecular mechanisms underlying these interactions remain poorly understood.

**Figure 5:**
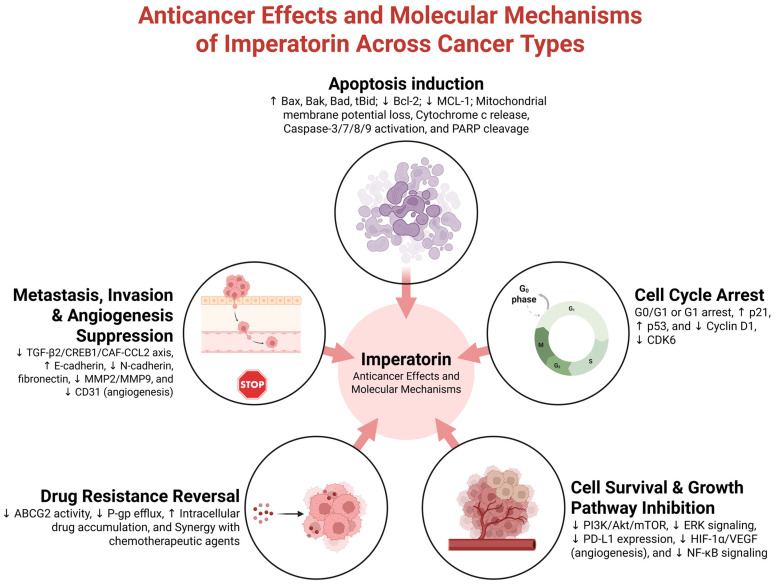
Anticancer mechanisms of imperatorin across diverse cancer models. Imperatorin demonstrates broad anticancer activity in liver, lung, glioblastoma, cervical, breast, esophageal, gastric, colon cancers, osteosarcoma, and others. Across in vitro and in vivo systems, imperatorin primarily induces mitochondrial-dependent apoptosis by modulating Bcl-2 family proteins, disrupting mitochondrial membrane potential, promoting cytochrome c release, and activating caspases. Additional mechanisms include inhibition of PI3K/Akt/mTOR, ERK, NF-κB, and PD-L1 signaling; induction of cell cycle arrest; suppression of angiogenesis and metastasis; reversal of multidrug resistance via ABC transporters; and synergistic enhancement of chemotherapeutic agents. Collectively, these findings support imperatorin as a multi-target anticancer compound with therapeutic potential across heterogeneous tumor contexts. Created in BioRender (https://biorender.com/zv0feod), last accessed on 16 December 2025.

Taken together, the current literature indicates that imperatorin has significant potential as an anticancer agent or as an adjunctive therapy. Its multitargeted mechanisms align well with the complex, heterogeneous nature of cancer, and its natural origin offers both structural diversity and a potentially favorable safety profile. However, realizing its translational potential will require substantial advances in pharmacological characterization, mechanistic elucidation, and preclinical validation. Figure 5 illustrates that imperatorin exhibits broad anticancer activity by inducing apoptosis, inhibiting survival pathways, and reversing drug resistance across multiple tumor types, as well as the related molecular mechanisms.

## 6. Future Research Perspectives

Future research on imperatorin should prioritize comprehensive pharmacokinetic, toxicological, and formulation studies, as these represent critical barriers to clinical application. Detailed analyses of absorption, distribution, metabolism, and excretion in multiple species are needed to clarify its bioavailability constraints and guide the development of optimized delivery systems. Novel formulations such as nanoencapsulation, lipid-based carriers, or prodrug strategies may enhance solubility, stability, and tissue-targeting properties, thereby improving therapeutic effectiveness. Equally important is the need for rigorous chronic toxicity and safety studies, including assessments of hepatotoxicity, cardiotoxicity, neurotoxicity, and reproductive toxicity, to establish a reliable safety margin.

Although actively targeted delivery systems (e.g., ligand-conjugated nanoparticles or receptor-specific carriers) have not yet been widely reported for imperatorin itself, several studies have explored formulation strategies designed to improve its delivery and bioavailability. For instance, imperatorin has been successfully encapsulated in ultradeformable lipid vesicles to enhance its transdermal permeation and skin penetration relative to free drug formulations, demonstrating improved flux and deeper tissue deposition across the stratum corneum barrier [175].

Similarly, liposomal hydrogel systems have been developed to improve topical delivery and retention of imperatorin, indicating formulation potential for controlled local delivery [176]. Additionally, imperatorin lipid microspheres formulated via nanoemulsion technology have been shown to significantly enhance systemic bioavailability and biological activity against MDA-MB-231 cells relative to the unformulated compound in animal models, suggesting promise for improved pharmacokinetics and therapeutic delivery [177].

These studies highlight the current focus on carrier-based approaches to enhance the delivery and absorption of imperatorin. While examples of actively targeted nanocarriers specifically designed for receptor-mediated targeting are still lacking, the formulation evidence indicates an emerging interest in improving imperatorin’s pharmacokinetic and tissue accessibility profiles.

At the mechanistic level, future investigations should expand beyond the predominantly apoptosis-focused frameworks to explore imperatorin’s effects on autophagy, ferroptosis, immunogenic cell death, metabolic reprogramming, and epigenetic regulation. Special attention should be given to how imperatorin shapes the tumor immune microenvironment, particularly given its ability to modulate PD-L1 expression, enhance γδ T cell cytotoxicity, and reduce pro-inflammatory signaling. These insights may illuminate opportunities to integrate imperatorin into immunotherapeutic strategies or combination regimens designed to overcome immune evasion.

Another critical avenue of research concerns drug resistance. Imperatorin’s capacity to modulate P-gp, ABCG2, and other efflux transporters suggests a potential role in preventing or reversing multidrug resistance, one of the most pressing obstacles in contemporary oncology. Elucidating the molecular basis of these interactions and exploring synergies with chemotherapeutics, targeted therapies, and radiotherapy may yield new strategies for managing refractory cancers. Future studies should also extend to patient-derived organoids, co-culture systems, and metastatic models that better recapitulate clinical complexity.

Given the centrality of natural biosynthesis to imperatorin’s availability, research into its plant biosynthetic pathways should continue to be strengthened. Fully functionally characterizing the elusive PT responsible for its formation, along with mapping downstream modifications and regulatory networks, may enable metabolic engineering strategies for sustainable, high-yield production. Such efforts would not only facilitate drug development but also support the generation of imperatorin analogs with improved potency, selectivity, and pharmacokinetics.

Ultimately, progressing imperatorin toward clinical evaluation will require integrated, multidisciplinary approaches that combine natural product chemistry, cancer biology, pharmacology, systems biology, and advanced preclinical modeling. By addressing the current gaps in mechanistic understanding, pharmacokinetic optimization, and safety validation, future studies will help determine whether imperatorin can transition from a promising phytochemical to a viable therapeutic candidate in oncology.

Although imperatorin has demonstrated promising anticancer activity in a range of in vitro and in vivo models, including induction of apoptosis, cell cycle arrest, and chemosensitization in multidrug-resistant cells, comprehensive medicinal-chemistry efforts aimed at systematically modifying its structure to improve potency, selectivity, and pharmacokinetic properties remain limited. Recent pharmacological reviews highlight that structural modification of imperatorin and related furanocoumarins represents a plausible strategy to enhance therapeutic efficacy and reduce side effects [44]. Still, detailed structure–activity relationship studies and optimized analogs specific to anticancer applications have not been widely reported to date. This gap underscores an essential opportunity for future investigation of imperatorin derivatives designed to improve drug-like properties and anticancer performance.

## Figures and Tables

**Table 1:** Plant sources of imperatorin, extraction methods, and reported contents or yields.

Plant Species	Plant Part	Extraction Method	Imperatorin Content/Yield	Reference
*A. officinalis* Hoffm.	Fruits	ASE (petroleum ether → methanol, 100 °C, 60 bar)	~19 mg/g	[48]
*P. sativa*	Fruits	ASE (petroleum ether → methanol, 100 °C, 60 bar)	>15 mg/g	[49]
*C. maxima*	Peel	Supercritical CO_2_ extraction (27.6 MPa, 50 °C, Ethanol)	~1.3 mg/g (95% efficiency)	[51]
*A. dahurica*	Herb	Solvent extraction + silica-gel column chromatography	~0.35 mg/g from 1 g material in very high purity (>99%)	[53]
*A. dahurica*	Herb	Counter-current chromatography	~30 mg purified imperatorin in very high purity (>98%)	[54]
*C. monnieri*	Fruits	Counter-current chromatography	>100 mg purified imperatorin at 98.2% purity	[55]

**Table 2:** Anticancer effects and underlying mechanisms of action of imperatorin across different cancer types in cellular and animal models.

Cell Line (s)/Animal Model (s)	IC_50_/EC_50_/Concentration and Duration	Effects Demonstrated	Mechanisms of Action	Reference
Liver Cancer				
In vitro: HepG2, Hep3B, PLC and Huh7.	In vitro: 2.5, 5, 10, 20, 40 and 80 μM; 24 and 48 h.	In vitro: ↑ cytotoxicity of cisplatin; reversion of the drug resistance in cisplatin-resistant HepG2 and cisplatin-resistant Huh7 cells. Synergistic effect: imperatorin + cisplatin induced apoptosis and mitochondrial membrane potential collapse more significant compared with either drug alone.	In vitro: ┴ expression of MCL-1.	[95]
In vitro: HepG2, Hep3B, and multidrug-resistant HepG2. In vivo: xenograft (inoculation of multidrug-resistant HepG2 cells) nude mouse model.	In vitro: 43.3 μM, 48 h (IC_50_ for HepG2 cells); 28.1 μM, 48 h (IC_50_ for multidrug-resistant HepG2 cells); 40, 80, 120, 160, and 200 μM; 48 h. In vivo: 50 mg/kg (intravenous injection) every 2 days, for a period of 14 days.	In vitro: ┴ multidrug-resistant HepG2 cell growth, ↑ multidrug-resistant HepG2 apoptosis. In vivo: ┴ multidrug-resistant HepG2 cell growth.	In vitro: induction of proteasomal degradation of MCL-1 (↓ MCL-1); ↑ expression of the death receptor protein Fas receptor; induction of Bak activation; ↑ Bax translocation from the cytoplasm to the mitochondrial fractions; activation of caspase-3, caspase-8, and caspase-9; induction of cytochrome c release; ↑ mitochondrial membrane potential disruption. In vivo: induction of MCL-1 degradation.	[96]
In vitro: HepG2. In vivo: xenograft (inoculation of HepG2 cells) nude mouse model.	In vitro: 101.2 μM, 24 h; 60.5 μM, 48 h and 22.4 μM, 72 h (IC_50_). In vivo: 50 or 100 mg/kg (oral administration) every day, for 14 consecutive days.	In vitro: ↓ cell proliferation, ↑ apoptosis. In vivo: ┴ tumor growth.	In vitro: activation of caspases and PARP cleavage; induction of cytochrome c release; ↑ mitochondrial membrane potential disruption; ↑ expression of the death receptor protein Fas receptor; ↑ protein expression patterns of p21 and p53; ↓ Bcl-2; ↑ Bax, Bad, and tBid. In vivo: the specific mechanisms have not been fully described.	[35]
Lung Cancer				
In vitro: CD133^+^ and CD133^−^ A549 and PC9 cells. In vivo: xenograft (injection of CD133^+^ A549 cells) nude BALB/c mouse model.	In vitro: 10 μM; 12 h. In vivo: 50 mg/kg (intraperitoneal injection) twice a week, for a period of 28 days.	In vitro: ↑ sensitivity of CD133^+^ cancer cells to γδ T cell-mediated cytotoxicity. Synergistic effect: imperatorin + γδ T cell therapy induced mitochondrial apoptosis. In vivo: ↓ tumor size. Synergistic effect: imperatorin + γδ T cell therapy significantly suppressed tumor growth.	In vitro: ↓ MCL-1 expression. In vivo: ┴ MCL-1 expression.	[97]
In vitro: H1975 and A549. In vivo: xenograft (injection of H1975 cells) BALB/c *nu/nu* mouse model and xenograft (injection of LLC cells) C57BL6/J mouse model.	In vitro: 9.64 ± 3.50 μM, 24 h and 5.28 ± 0.50 μM, 48 h (IC_50_ for H1975 cells); 18.20 ± 1.35 μM, 24 h and 14.17 ± 3.02 μM, 48 h (IC_50_ for A549 cells). In vivo: 20 or 40 mg/kg (intraperitoneal injection) daily for 21 days.	In vitro: ┴ cell growth. In vivo: ↓ tumor size, volume, and weight.	In vitro: modulation of the PI3K/Akt and PD-L1 pathways. In vivo: ┴ PI3K/Akt pathway; downregulation of PD-L1 expression in tumor tissues.	[32]
In vitro: NCI-H23, NCI-H292, and A549.	In vitro: 0.1, 0.5, 1, 5, and 10 µg/mL; 12 and 24 h.	In vitro: ↑ cell apoptosis after detachment, ┴ anchorage-independent cell growth, ↑ cell sensitization to anoikis.	In vitro: ↑ p53 protein level; downregulation of MCL-1 protein; upregulation of Bax.	[98]
Glioblastoma				
In vitro: T98G.	In vitro: 25, 50, and 100 µM; 24 and 48 h.	In vitro: ↑ apoptosis. Synergistic effect: imperatorin + quercetin induces apoptosis more efficiently than either drug alone.	In vitro: ↓ HSP27 and HSP72 expression; ↑ caspase-3 and caspase-9 activity.	[99]
Cervical Cancer				
In vitro: HeLa.	In vitro: 5, 20, 50, 100 and 150 µM; 8, 12 and 24 h.	In vitro: pro-apoptotic and anti-inflammatory effects.	In vitro: ┴ TNF-α-induced expression of NF-κB target genes; ┴ NF-κB activation (suppression of TNF-α-induced IKKα/β phosphorylation, IκB phosphorylation and degradation, and NF-κB p65 nuclear translocation); downregulation of TNF-α-induced activation of PI3K/Akt; ┴ TNF-α-induced ROS generation.	[40]
Breast Cancer				
In vitro: MCF-7.	In vitro: 0.1, 0.25, 0.5, 0.75, 1 and 1.5 µM; 24 h.	In vitro: ↓ cell viability. Synergistic effect: imperatorin attenuates cell proliferation and promotes apoptosis when combined with radiotherapy or hyperthermia.	Synergistic effect: ↑ Bax, caspase-3, caspase-8, and caspase-9; ↓ Bcl-2.	[100]
Esophageal Cancer				
In vitro: KYSE30 and KYSE150. In vivo: xenograft (injection of luciferase-expressing KYSE150 cells) nude mouse model and xenograft (injection of luciferase-expressing EC9706 cells) NCG mouse model.	In vitro: 40 and 80 µM; 24 h. In vivo: 25 or 50 mg/kg (oral administration) 2–3 times a week.	In vitro: ┴ cell invasive potential, ┴ tumor angiogenesis. In vivo: ┴ tumor metastasis (lungs, liver, kidneys, and spleen), ┴ tumor angiogenesis.	In vitro: ↓ TGF-*β*2 expression; ┴ transcriptional activity of CREB1; ┴ ERK signaling; ┴ CAFs-secreted CCL2; ↑ E-cadherin expression; ↓ expression levels of fibronectin, N-cadherin, MMP2, and MMP9. In vivo: ┴ transcriptional activity of CREB1; ┴ TGF-*β*2-ERK signaling; ┴ fibroblasts-secreted CCL2.	[101]
Gastric Cancer				
In vitro: SGC-7901.	In vitro: 62.6 µM (IC_50_).	In vitro: ┴ cell growth, ↑ apoptosis; promotion of DNA damage, cell shrinkage, and distortion of normal cellular structures.	In vitro: downregulation of PI3K/Akt/mTOR signaling proteins; induction of sub-G1 cell cycle arrest.	[102]
Colon Cancer				
In vitro: HT-29.	In vitro: 239 µM, 24 h; 101 µM, 48 h and 78 µM, 72 h (IC_50_).	In vitro: ┴ cell proliferation and viability, ↑ apoptosis.	In vitro: upregulation of p53; activation of caspase-3 and caspase-7; ↑ Bax/Bcl-2 ratio expression; induction of cell cycle arrest at G1; ↑ ROS levels.	[34]
In vitro: HCT116. In vivo: xenograft (injection of HCT116 cells) nude BALB/c mouse model.	In vitro: 10, 50, 100, and 150 µM; 12 and 24 h. In vivo: 50 or 100 mg/kg (oral administration) 3 times a week, for a period of 40 days.	In vitro: ┴ cell proliferation. In vivo: ┴ tumor growth and tumor angiogenesis.	In vitro: ↓ HIF-1α protein synthesis and levels; ┴ VEGF and EPO mRNA expression; induction of cell cycle arrest at G1; downregulation of mTOR/p70S6K/4E-BP1 and MAPK signaling pathways. In vivo: ┴ mTOR/p70S6K/4E-BP1 and MAPK signaling pathways; ┴ HIF-1α protein level; ↓ VEGF and CD31 expression.	[37]
Osteosarcoma				
In vitro: U2OS and 143B. In vivo: xenograft (injection of 143B cells) nude BALB/c mouse model.	In vitro: 131.4 μM, 24 h and 116.3 μM, 48 h (IC_50_ for U2OS cells); 118.7 μM, 24 h and 90 μM, 48 h (IC_50_ for 143B cells). In vivo: 5 mg/kg (intraperitoneal injection) every other day, 5 times in total.	In vitro: ┴ cell proliferation, migration, and invasion. In vivo: ┴ tumor growth.	In vitro: ↑ autophagy (↑ ATG1, ATG5 and LC3B); induction of G0/G1 cell cycle arrest (↓ cyclin D1 and CDK6); upregulation of PTEN and p21 expression; ↓ phosphorylation of Akt and mTOR. In vivo: ↑ PTEN- and LC3-positive cells and p-Akt- and CDK6-negative cells in tumor tissues.	[103]
Multicancer Studies				
In vitro: KB-3-1 and KB-V1, ovarian cancer (OVCAR-8 and NCI-ADR-RES), lung cancer (H460 and H460-MX20), colon cancer (S1 and S1-MI-80).	In vitro: 1, 2, 5, and 10 µM (1–100 µM); 48 and 72 h.	In vitro: reversion of ABCG2-mediated multidrug resistance in cancer cells, potentiation of topotecan-induced apoptosis in ABCG2-overexpressing cancer cells, ↓ cell viability.	In vitro: ┴ drug efflux function of ABCG2; ↑ intracellular accumulation of ABCG2 substrate pheophorbide A.	[104]
In vitro: leukemia (K562 and doxorubicin-resistant K562), ovarian cancer (A2780 and taxol-resistant A2780).	In vitro: 2.78, 2.8, 5.56, and 11.10 µM; 70, 100, and 120 min.	In vitro: restriction of glycolysis and glutamine metabolism of doxorubicin-resistant K562 cells, ↑ cytotoxicity of doxorubicin and taxol; reversion of the drug resistance in doxorubicin-resistant K562 cells and taxol-resistant A2780 cells.	In vitro: ↑ intracellular Rho123 accumulation; ↓ efflux activity of P-gp.	[105]
In vitro: liver cancer (SNU 449) and colon cancer (HCT-15).	In vitro: 7.4, 74, 370, and 740 nmol/mL; 24 h and 4 days.	In vitro: ┴ cell growth, ↑ apoptosis.	In vitro: induction of cell cycle arrest at G1-SubG1; ↓ Bcl-2/Bax ratio.	[106]
In vitro: rhabdomyosarcoma (TE671), lung cancer (A549, H2170 and H1299), larynx cancer (RK33 and RK45).	In vitro: 111.2 µM (IC_50_ for TE671 cells), 67.8 µM (IC_50_ for RK33 cells), 1–200 µM; 24, 48 and 72 h.	In vitro: ↓ cell viability and proliferation, ↑ apoptosis (these effects were more pronounced in TE671 and RK33 cells).	In vitro: ↑ cleaved caspase-3; induction of cell cycle arrest at G1; modulation of p21 and cyclin D1 expression.	[107]
In vitro: cervical cancer (HeLa) and Hep-2.	In vitro: 50 and 100 µM; 24 and 48 h.	In vitro: ↓ cell viability, ↑ apoptosis. Synergistic effect: imperatorin + quercetin induced apoptosis remarkably stronger than each drug alone.	In vitro: ↓ HSP72 expression; ↑ caspase activity.	[108]

Note: Various symbols (↑, ↓, and ┴) indicate an increase, a decrease, and an inhibition in the obtained variables, respectively.

## Data Availability

Data sharing not applicable.

## Abbreviations

The following abbreviations are used in this manuscript:

4CL	4 Coumarate CoA ligase
4E-BP1	Eukaryotic initiation factor 4E-binding protein 1
ABCG2	ATP-binding cassette subfamily G member 2
ACR	Accessible chromatin regions
ADMET	Absorption, Distribution, Metabolism, Excretion, and Toxicity
Akt	Protein Kinase B
ARE	Antioxidant response element
ASE	Accelerated solvent extraction
ATG	Autophagy-related protein
AUC	Area under the curve
Bad	Bcl-2-associated death promoter
Bak	Bcl-2 homologous antagonist/killer
Bax	Bcl-2-associated X protein
Bcl-2	B-cell lymphoma 2
CAF	Cancer-associated fibroblast
C4H	Cinnamate 4-hydroxylase
CCL2	C-C motif chemokine ligand 2
CDK6	Cyclin-dependent kinase 6
COX-2	Cyclooxygenase-2
CREB1	cAMP response element-binding protein 1
DMAPP	Dimethylallyl pyrophosphate
DNA	Deoxyribonucleic acid
EGFR	Epidermal growth factor receptor
EPO	Erythropoietin
ERK	Extracellular signal-regulated kinase
GC–MS	Gas chromatography–mass spectrometry
GSH	Glutathione
HBV	Hepatitis B virus
HCC	Hepatocellular carcinoma
HCV	Hepatitis C virus
HF-LPME	Hollow fiber liquid-phase microextraction
HIF-1α	Hypoxia-inducible factor 1 alpha
HO-1	Heme oxygenase-1
HPLC–ESI-MS	High-performance liquid chromatography–electrospray ionization–mass spectrometry
HPLC-UV	High-performance liquid chromatography with ultraviolet detection
HPV	Human papillomavirus
HSP	Heat shock protein
IKKα/β	IκB kinase alpha/beta
IL	Interleukin
iNOS	Inducible nitric oxide synthase
IκBα	Inhibitor of kappa B alpha
JAK	Janus kinase
JNK	c-Jun N-terminal kinase
LC3	Microtubule-associated protein 1A/1B-light chain 3
LC–MS	Liquid chromatography–mass spectrometry
MAPK	Mitogen-activated protein kinase
MCL-1	Myeloid cell leukemia 1
MDA	Malondialdehyde
MGMT	O-methylguanine-DNA methyltransferase
MMP	Matrix metalloproteinase
mRNA	Messenger ribonucleic acid
mTOR	Mammalian target of rapamycin
NASH	Non-alcoholic steatohepatitis
NF-κB	Nuclear factor kappa B
NQO-1	NAD(P)H quinone oxidoreductase 1
NSCLC	Non-small cell lung cancer
Nrf2	Nuclear factor erythroid 2–related factor 2
PAL	Phenylalanine ammonia lyase
PARP	Poly (ADP-ribose) polymerase
PDE4	Phosphodiesterase-4
PD-L1	Programmed death-ligand 1
p-Akt	Phosphorylated Protein Kinase B
PI3K	Phosphatidylinositol 3-kinase
PKA	Protein Kinase A
PT	Prenyltransferase
PTEN	Phosphatase and Tensin Homolog
P-gp	P-glycoprotein
ROS	Reactive oxygen species
SCLC	Small cell lung cancer
SFE	Supercritical fluid extraction
SOD	Superoxide dismutase
STAT	Signal transducer and activator of transcription
TGF-β2	Transforming growth factor beta 2
TNF-α	Tumor necrosis factor alpha
tBid	Truncated BH3-interacting domain death agonist
UGT	UDP-glucosyltransferase
VEGF	Vascular endothelial growth factor

## Appendix A

A comprehensive literature search was conducted across several major scientific databases and content aggregators, including PubMed, Scopus, Web of Science, Scielo, Embase, and Google Scholar. This review critically evaluates the anticancer potential of imperatorin, a bioactive furanocoumarin increasingly recognized for its diverse pharmacological properties. Despite the expanding body of preclinical evidence published in recent years, no comprehensive review has thoroughly synthesized the anticancer mechanisms and therapeutic prospects of imperatorin. Therefore, a broad, methodical search strategy was used to capture all relevant experimental studies.

Keywords and controlled vocabulary terms were selected to maximize search sensitivity. Search terms included “imperatorin,” “cancer,” “tumor,” “cancer cell lines,” “animal models,” “breast cancer,” “lung cancer,” “colorectal cancer,” “gastric cancer,” “leukemia,” “metastasis,” “apoptosis,” “cell cycle arrest,” “cell proliferation,” “ROS,” “MAPK,” “PI3K/Akt,” “NF-κB,” “mTOR,” “inflammation,” “DNA damage,” “chemoprevention,” and “anticancer mechanisms.”

Because imperatorin has not yet advanced to oncology clinical trials, the inclusion criteria focused exclusively on in vitro and in vivo experimental research investigating its anticancer properties. Eligible studies examined the effects of imperatorin on cancer cell viability, apoptosis induction, migration and invasion, tumor growth in animal models, treatment regimens, administration routes, and modulation of molecular pathways associated with carcinogenesis.

Studies were excluded if they met any of the following conditions: review articles, meta-analyses, editorials, letters, conference abstracts, non-experimental papers, studies not involving imperatorin as the primary intervention, or studies unrelated to cancer models. No publication-year restrictions were applied to ensure a comprehensive dataset; however, studies lacking methodological clarity, experimental rigor, or transparent reporting were excluded.

Data extraction was performed independently by two experienced reviewers using a standardized protocol consistent with comprehensive review procedures. Extracted information included study design, experimental model, characteristics of imperatorin treatment (concentration, exposure time, delivery method), and key outcomes. The PICO framework—Population, Intervention, Comparison, Outcome—guided the extraction and evaluation process, while study quality was assessed based on experimental design and reproducibility. This review was conducted in accordance with the PRISMA principles for rigor and quality [94].

A qualitative synthesis approach was used to summarize and compare findings across studies. Extracted data were organized into a table detailing the anticancer activities of imperatorin, reported mechanisms of action, and evidence supporting its potential role as an anticancer or chemopreventive agent. This review also identifies research gaps, highlights methodological limitations in current investigations, and proposes future directions to advance the translational potential of imperatorin in oncology.
